# Supplementary material for: Generative language models on nucleotide sequences of human genes
Source: Sci Rep. 2024 Sep 27;14:22204. doi: 10.1038/s41598-024-72512-x (PMC11437190; doi:10.1038/s41598-024-72512-x)
Supplement: Supplementary file 1 — Supplementary Information. [file 41598_2024_72512_MOESM1_ESM.pdf]

## Appendix

**Table S1.** Hyperparameter experiments for rnn model

| Hyperparameters | Validation Perplexity |
|---|---|
| Initial:<br>- EMBED_DIM = 256<br>- LSTM_DIM = 256<br>- NUM_LAYERS = 2 | 3.5257 |
| Change:<br>NUM_LAYERS = 1 | 3.5951 |
| Change:<br>NUM_LAYERS = 3 | 3.4770 |
| Change:<br>EMBED_DIM = 128 | 3.4995 |
| Change:<br>LSTM_DIM = 128 | 3.6067 |
| Change:<br>EMBED_DIM = 512 | 3.5441 |
| Change:<br>LSTM_DIM = 512 | 3.4399 |
| Change:<br>NUM_LAYERS = 4 | 3.4637 |
| Change:<br>NUM_LAYERS = 4<br>EMBED_DIM = 128<br>LSTM_DIM = 512 | 3.3305 |
| Change:<br>NUM_LAYERS = 4<br>LSTM_DIM = 512 | **3.2726 (CHOSEN)** |
| Change:<br>NUM_LAYERS = 5<br>LSTM_DIM = 512 | 3.3610 |
| Change:<br>NUM_LAYERS = 4<br>LSTM_DIM = 1024 | 3.3656 |

**Table S2.** Hyperparameter experiments for transformer model

| Hyperparameters | Validation Perplexity |
|---|---|
| Initial:<br>- EMBED_DIM = 256<br>- FEED_FORWARD_DIM = 256<br>- NUM_LAYERS = 2<br>- NUM_HEADS = 3 | 3.7018 |
| Change:<br>NUM_LAYERS = 3 | 3.6932 |
| Change:<br>NUM_HEADS = 4 | 3.7011 |
| Change:<br>EMBED_DIM = 512 | 3.7189 |
| Change:<br>FEED_FORWARD_DIM = 512 | 3.7064 |
| Change:<br>NUM_LAYERS = 4 | **3.6880 (CHOSEN)** |
| Change:<br>NUM_LAYERS = 5 | 3.6903 |
| Change:<br>EMBED_DIM = 128 | 3.7022 |
| Change:<br>FEED_FORWARD_DIM = 128 | 3.7095 |
| Change:<br>NUM_LAYERS = 4<br>NUM_HEADS = 4 | 3.6959 |
